# Kidney Cancer Prediction Empowered with Blockchain Security Using Transfer Learning

**DOI:** 10.3390/s22197483

**Published:** 2022-10-02

**Authors:** Muhammad Umar Nasir, Muhammad Zubair, Taher M. Ghazal, Muhammad Farhan Khan, Munir Ahmad, Atta-ur Rahman, Hussam Al Hamadi, Muhammad Adnan Khan, Wathiq Mansoor

**Affiliations:** 1 Riphah School of Computing and Innovation, Riphah International University Lahore Campus, Lahore 54000, Pakistan; 2 Faculty of Computing, Riphah International University, Islamabad 45000, Pakistan; 3 Center for Cyber Security, Faculty of Information Science and Technology, Universiti Kebangsaan Malaysia (UKM), Bangi 43600, Selangor, Malaysia; 4 College of Computer and Information Technology, American University in the Emirates, Dubai Academic City, Dubai 503000, United Arab Emirates; 5 Department of Forensic Sciences, University of Health Sciences, Lahore 54000, Pakistan; 6 School of Computer Science, National College of Business Administration & Economics, Lahore 54000, Pakistan; 7 Department of Computer Science, College of Computer Science and Information Technology (CCSIT), Imam Abdulrahman Bin Faisal University (IAU), P.O. Box 1982, Dammam 31441, Saudi Arabia; 8 College of Engineering and IT, University of Dubai, Dubai 14143, United Arab Emirates; 9 Department of Software, Gachon University, Seongnam 13120, Korea

**Keywords:** kidney cancer, transfer learning, IoMT, deep learning, blockchain

## Abstract

Kidney cancer is a very dangerous and lethal cancerous disease caused by kidney tumors or by genetic renal disease, and very few patients survive because there is no method for early prediction of kidney cancer. Early prediction of kidney cancer helps doctors start proper therapy and treatment for the patients, preventing kidney tumors and renal transplantation. With the adaptation of artificial intelligence, automated tools empowered with different deep learning and machine learning algorithms can predict cancers. In this study, the proposed model used the Internet of Medical Things (IoMT)-based transfer learning technique with different deep learning algorithms to predict kidney cancer in its early stages, and for the patient’s data security, the proposed model incorporates blockchain technology-based private clouds and transfer-learning trained models. To predict kidney cancer, the proposed model used biopsies of cancerous kidneys consisting of three classes. The proposed model achieved the highest training accuracy and prediction accuracy of 99.8% and 99.20%, respectively, empowered with data augmentation and without augmentation, and the proposed model achieved 93.75% prediction accuracy during validation. Transfer learning provides a promising framework with the combination of IoMT technologies and blockchain technology layers to enhance the diagnosing capabilities of kidney cancer.

## 1. Introduction

Kidney cancer incidence is increasing yearly, reaching 0.1550% (95 percent CI: 0.155–0.163%) in 2018. The dependent 5-year overall survival rate was just 85.8% (95 percent CI: 85.5–86%), demonstrating that this illness has a significant death risk. Clear cell renal cell carcinoma is the most prevalent and lethal subtype of renal carcinoma, accounting for roughly 75% of all renal carcinomas [1,2]. Tumor metastasis is the leading cause of mortality in KIRC patients [3]. Early-stage kidney cancer has no clear clinical signs, and 26–31% of patients have already metastasized to distant before they are detected [4,5,6]. Patients with KIRC who have had their local tumors eliminated by prostatectomy are still at significant risk of metastasis and recurrence, and they are resistant to chemotherapy and radiation, resulting in a dismal prognosis [7,8,9]. As a result, early recognition and diagnosis of this condition are critical. Understanding the primary gene factors for growth may also aid in the development of novel therapeutics [10].

Epidemiology is linked to the development of chronic kidney disease and many other clinical characteristics. In general, nephrologists employ blood tests and urine tests to confirm the use of CKD [11]. Genetics, diabetes, obesity, and age are all risk factors for chronic kidney disease. The blood test determines how efficiently the kidneys filter blood to eliminate creatinine, a typical waste product of muscle breakdown [12]. Rather, the urine sample will demonstrate that the protein remains in frequent urination and that albumin is a component of the blood that is not ordinarily transported to the urine via the kidney filter [13]. When a urinalysis reveals that protein is being generated, it suggests that the renal filters have been compromised and may indicate chronic renal disease.

**Figure 1:**
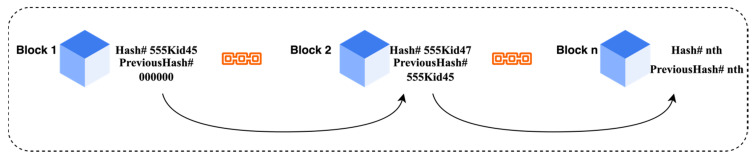
Blockchain architecture.

For health care systems, transfer learning poses a number of security risks. Attackers can simply obtain access to unified networks and steal data. The main disadvantage of hacking efforts on various health care networks and patient records is that they might endanger patients’ lives by distributing phishing and spam emails to them. Most network systems in health care institutions are centralized, and their networks are frequently the major targets of hacker assaults. All of these problems are caused by centralized networks. All of these difficulties may be readily handled by utilizing blockchain-secured cloud infrastructure. Satoshi Nakamoto created blockchain in 2008, comprising a time-stamped collection of numerous hackers’ evidence files that are safeguarded by a network of separate networks. Figure 1 depicts blockchain technology’s architecture. It is a straightforward cryptographically linked set of blocks. Blockchain technology has several responsibilities, such as transparency, decentralization, and rigidity. These three roles provided them with opportunities to link with embedded devices, centralized networks, virtual currencies, etc.

## 2. Literature Review

The authors of [14] attempted to categorize kidney cancer subtypes using miRNA sequencing data for substituent component analysis. The purpose of categorizing a given miRNA dataset into kidney cancer subtypes is to extract discriminating miRNA characteristics and long-term memory, which is represented by a recurrent neural network. The substituent component analysis approach was used to select the 35 most biased miRNA data sets. This group of miRNAs enables long-term memory to categorize kidney cancer miRNAs into five subtypes with an average accuracy of roughly 95% and Matthew’s correlation coefficient values of approximately 0.92 in 10 randomized five-fold trials, which is extremely close.

A deep neural network was used in [15] to discover and classify novel kidney histological abnormalities. They demonstrate that DNN-based machine learning provides a strong and generalized performance on a variety of histology image processing tasks. The neural network retrieved and classified quantitative picture information to categorize the differences between mice of various genotypes. The separation of the semi and the genotype of the animal based on quantitative imaging parameters demonstrated good performance. These traits were not discovered in a systematic pathological examination on the Internet of Medical Things platform.

A hybrid neural network was proposed in [16] to detect kidney cancer. To properly incorporate the data in electronic health records, they represented the specific prediction issue as a two-class function. The recurrent neural network was trained in this study [17] to predict the onset of acute renal damage. The input sequence will be determined by the recurrent neural network so that answers to earlier portions of the series are examined at a particular point in the sequence. The recurrent neural network predicted the likelihood of acute kidney damage in clinical parameter sequences. 

They proposed a hybrid neural network [18] that combines bidirectional long-term memory with automated encoding networks. The authors claimed that they built a data collection based on much of the raw data from electronic health records. There are 36,132 reports of hypertensive patients in the collection. The results of the testing demonstrate that the suggested neural model for the task achieves 89.7% accuracy. The model was provided with an artificial neural network, and the results of a model analysis revealed that the performance of the recommended neural model is essential for the data set created.

The model correctly predicted that around 56% of all occurrences of acute kidney injury would advance to diagnostic techniques [19] within a timeframe of up to 48 h at the location of injury for each of the established severity categories of acute renal injury. With a 33% accuracy, the procedure was chosen. That is, acute [20] renal damage was present in one of the three anticipated instances, whereas the prognosis in the other two cases was mistakenly reported. Additional testing revealed that in patients with chronic kidney disease, 57% of the estimations were incorrect.

An assembling multi-stage deep learning strategy for kidney tumor segmentation was introduced [21]. To combine the previous phases’ prediction outputs, a combination process was performed to find the variance between different models in [22]. The average Dice score for kidney and pediatric malignancies among 90 unnamed test cases was 0.96 and 0.74, respectively. The findings are promising, but they might be enhanced by including a better knowledge of benign cysts, which generally limit tumor division [23] on the Internet of Medical Things platform [24]. The outcomes are dramatically decreased, as stated in this research. In comparison [25] to the attempted batches of 8 to 16, the batch normalization qualities were better utilized with the comparatively high batch size. The 42 samples’ outcomes improved between examinations.

There are numerous limitations in previous studies of machine learning and deep learning techniques, the major thing being the absence of patient data security, which is very crucial and alarming for the health sector. Table 1 shows the previous studies and their limitations.

Therefore, in this study, the proposed model used different deep learning techniques empowered with transfer learning to diagnose kidney cancer. The proposed model prototype used IoMT technology and blockchain security technologies to enhance the quality and prediction of kidney disease. The major contributions of this study are given below:

- IoMT-based proposed framework.
- The proposed model used blockchain technology for patients’ data security.
- The proposed model used various deep learning techniques empowered with transfer learning with the help of various parameters to predict kidney cancer.
- Numerous statistical matrixes were used to check the performance and reliability of the proposed framework.

## 3. Materials and Methods

**Figure 2:**
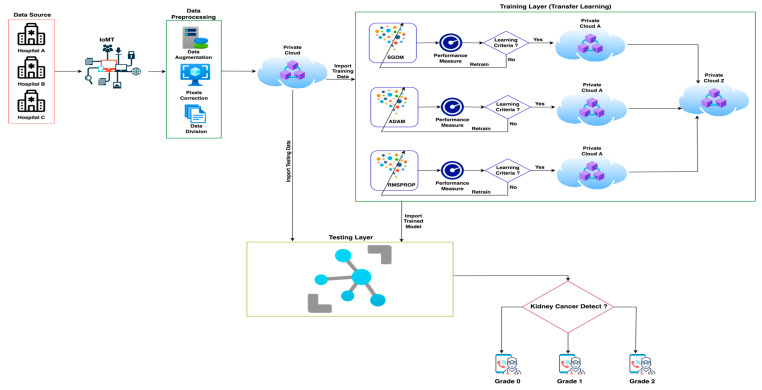
The proposed IoMT-based model for the prediction of kidney cancer empowered with blockchain security using transfer learning.

Figure 2 shows the total overview of the proposed methodology of IoMT-based prediction of kidney cancer empowered with blockchain security for patient and clinical data security using transfer learning. At the initial stage of the proposed framework, data were collected from different hospitals using Internet of Medical Things technology and sent to the data preprocessing layer. Data preprocessing depicts data augmentation and pixel correction. The proposed framework used data augmentation techniques to balance the data samples to overcome the limitations of imbalanced data, and for pixel correction, the proposed model used different image processing techniques to enhance the data sample quality for better prediction accuracy. After data preprocessing, the proposed framework divides data into training and testing and stores it in a secure private cloud on the blockchain. In the second phase, the training layer imports data from the private cloud and provides Stochastic Descent Gradient Momentum (SGDM), Adaptive Momentum Estimation (ADAM) and Root Mean Square Propagation (RMSPROP) deep learning algorithms to train the data samples.

After training the data samples, the proposed framework measures the learning criteria of all models. If models reach the learning criteria, then all training models are stored separately in their secured, private blockchain clouds; otherwise, the models are retrained. After training all of the models, the best-trained model is imported to the secure, private blockchain cloud Z for the further testing process. In the final phase, i.e., the testing phase, the following steps are performed: (1) import the best-trained model from private cloud Z and import the testing data samples from a private cloud of data; (2) apply the testing techniques and predict the kidney cancer (kidney cancer data samples are categorized into three predictive classes: grade 0, grade 1, and grade 2); (3) apply various statistical matrixes, e.g., Classification Accuracy (CA), Miss-Classification Rate (MCR), sensitivity, specificity, f1-score, Positive Predicted Value (PPV), Negative Predicted Value (NPV), False Positive Rate (FPR), False Negative Rate (FNR), Likelihood Positive Ratio (LPR), Likelihood Negative Ratio (LNR) and Fowlkes Mallows Index (FMI), to check the performance of the proposed framework (all equations are explained below) [28,29,30,31,32,33,34]. After the kidney cancer prediction, patients can consult with doctors for early therapy. Table 2 depicts the descriptive pseudocode of the proposed framework for the prediction of kidney cancer.

(1) ∂p=ℵpζp

where ∴ ℵ is for predicted class, and ζ is for true class.

(2) χp=∑h=13(ℵpζh≠p)

(3) ςp=∑h=13(ℵh≠pζp)

(4) ϱp=∑h=13(ℵh≠pζh≠p)

(5) CA=ℵpζp+∑h=13(ℵpζh≠p) ℵpζp+∑h=13(ℵpζh≠p)+∑h=13(ℵh≠pζp)+∑h=13(ℵh≠pζh≠p) × 100

(6) CMR=100 − (ℵpζp+∑h=13(ℵpζh≠p) ℵpζp+∑h=13(ℵpζh≠p)+∑h=13(ℵh≠pζp)+∑h=13(ℵh≠pζh≠p)×100)

(7) Sensitivity=ℵpζp ℵpζp+∑h=13(ℵh≠pζh≠p)×100

(8) Specificity=∑h=13(ℵpζh≠p)∑h=13(ℵpζh≠p)+∑h=13(ℵh≠pζp)×100

(9) F1-Score=2 ℵpζp 2 ℵpζp+∑h=13(ℵh≠pζp)+∑h=13(ℵh≠pζh≠p) ×100

(10) PPV=ℵpζp ℵpζp+∑h=13(ℵh≠pζp)×100

(11) NPV=∑h=13(ℵpζh≠p)∑h=13(ℵpζh≠p)+∑h=13(ℵh≠pζh≠p)×100

(12) FPR=100−(∑h=13(ℵpζh≠p)∑h=13(ℵpζh≠p)+∑h=13(ℵh≠pζp)×100)

(13) FNR=100 − (ℵpζp ℵpζp+∑h=13(ℵh≠pζh≠p)×100)

(14) LPR=ℵpζp ℵpζp+∑h=13(ℵh≠pζh≠p)×100100−(∑h=13(ℵpζh≠p)∑h=13(ℵpζh≠p)+∑h=13(ℵh≠pζp)×100)

(15) LNR=100−(ℵpζp ℵpζp+∑h=13(ℵh≠pζh≠p)×100)∑h=13(ℵpζh≠p)∑h=13(ℵpζh≠p)+∑h=13(ℵh≠pζp)×100

(16) FMI=(ℵpζp ℵpζp+∑h=13(ℵh≠pζh≠p)×100)×(ℵpζp ℵpζp+∑h=13(ℵh≠pζp)×100)

## 4. Data Set

**Figure 3:**
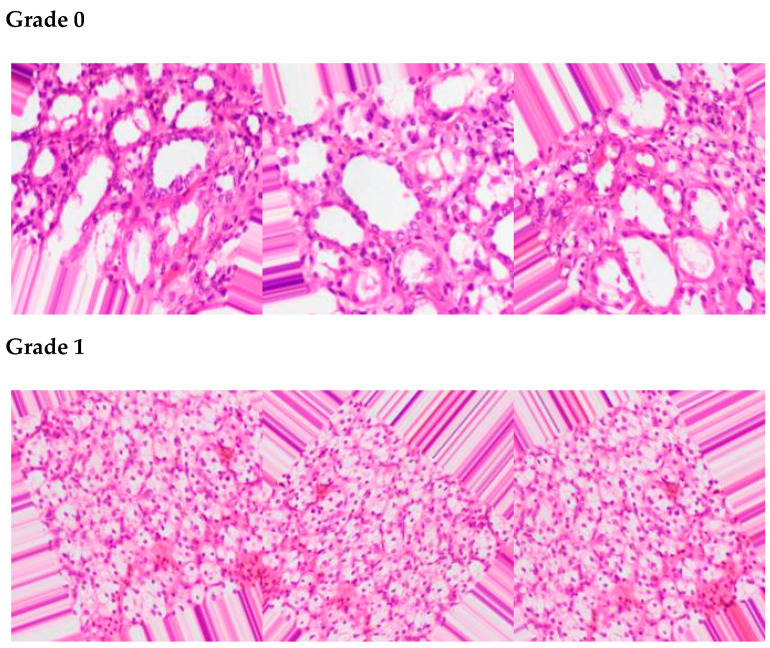

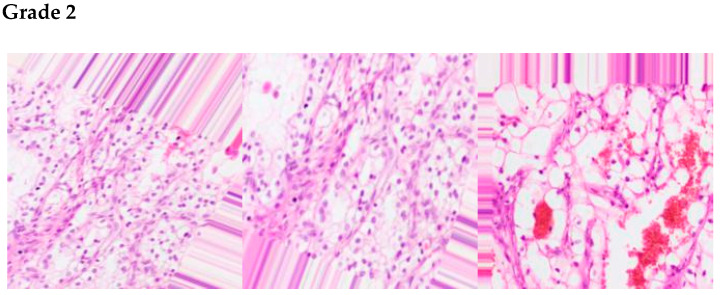
Data samples from grade 0, grade 1, and grade 2 classes.

The proposed framework acquired data from an online source [35] for the prediction of kidney cancer. The dataset consists of three classes of kidney cancer: grade 0, grade 1, and grade 2, and each class carries 1100 data samples of kidney cancer. The total instances of the dataset are 3300 after data augmentation from the proposed framework. The proposed model augments the dataset using position augmentation techniques, such as scaling, flipping, and color augmentation (e.g., contrast and brightness enhancement). Figure 3 depicts some samples from each class of the dataset.

## 5. Simulation and Results

In this article, IoMT-based deep learning techniques empowered with transfer learning and blockchain security were used to predict kidney cancer in its early stages for better therapy. MATLAB 2021 was used for simulation purposes. For training and testing simulation purposes, a MacBook Pro 2017 with 16GB RAM and 512GBSSD was used for the proposed framework. The proposed model used a dataset in two parts: training and testing of 70% and 30%, respectively. In the data preprocessing stage, the proposed framework applied data augmentation techniques to balance the data in all predicted classes and pixel correction techniques to enhance the images for better prediction results. Various deep learning techniques empowered with transfer learning were used to train the models with the help of kidney cancer data samples, and testing techniques were applied to predict the cancer at each grade level. The proposed framework used various statistical matrixes to check the performance of all models and choose the best model for prediction. All matrix equations have been mentioned before.

The proposed model customized the last three layers for analysis and prediction of kidney cancer. The proposed model customizes the fully connected layer according to the output size of instances. The softmax layer precisely observes and detects the boundaries of input instances and constitutes the convolutional layer, and the convolutional layer extracts the features from input instances using the gabber filter. Every convolutional layer uses a rectified linear unit activation function to activate the neurons and sends them to a fully connected layer for a weighted sum. Therefore, the proposed method applied this customized AlexNet model to predict kidney cancer.

Table 3 shows the overall training performance of all deep learning models empowered with transfer learning. The proposed framework tunes all models at 500 iterations, 0.001 learning rate, and 20 epochs. Therefore, SGDM performs well above all training models and achieves the highest classification accuracy and miss-classification rate (99.8%, 0.2%), respectively.

**Figure 4:**
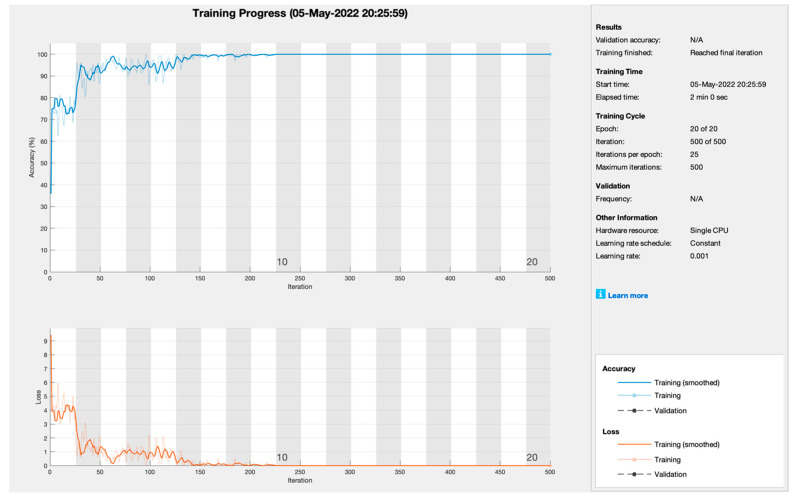
Training progress of SGDM empowered with AlexNet to predict kidney cancer.

Figure 4 depicts the training progress of stochastic gradient descent momentum empowered with transfer learning. The proposed framework sets the learning rate at 0.001 at 20 epochs with 25 iterations per epoch to train the model. As Figure 3 shows, training progress is very smooth and converges at the 7th epoch. Therefore, the training model SGDM achieves the highest training prediction accuracy and loss rate of 99.8% and 0.2%, respectively.

**Figure 5:**
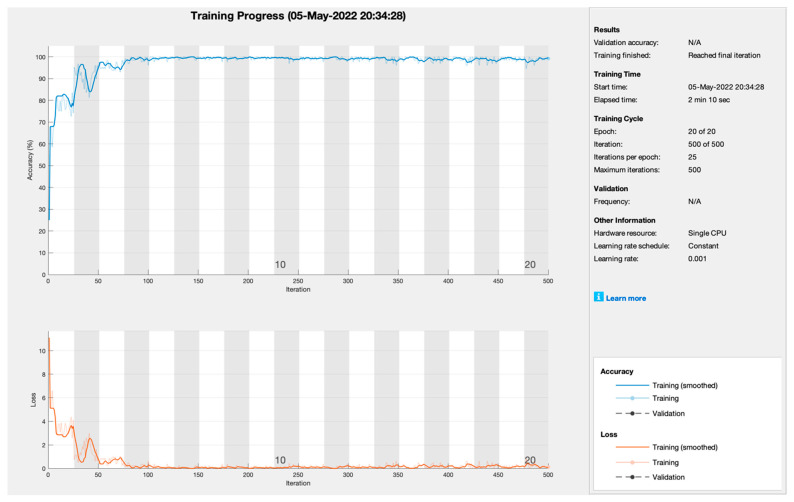
Training progress of ADAM empowered with AlexNet to predict kidney cancer.

Figure 5 depicts the training progress of adaptive moment estimation empowered with transfer learning. The proposed framework set the learning rate at 0.001 at 20 epochs with 25 iterations per epoch to train the model. As Figure 4 shows, training progress is very smooth and converges at the 5th epoch but has distortion till the 20th epoch. Therefore, the training model ADAM achieves the highest training prediction accuracy of 99.00% and 1.00% loss rate, respectively.

**Figure 6:**
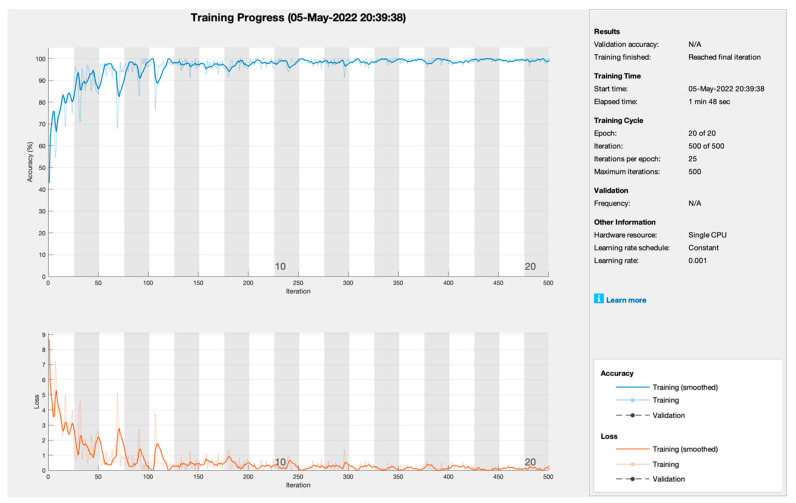
Training progress of RMSPROP empowered with AlexNet to predict kidney cancer.

Figure 6 depicts the training progress of root mean square propagation empowered with transfer learning. The proposed framework set the learning rate at 0.001 at 20 epochs with 25 iterations per epoch to train the model. As Figure 5 shows, training progress is not very smooth and did not converge properly till the 20th epoch. Therefore, the training model RMSPROP achieves a training prediction accuracy of 98.98% and 1.02% loss rate, respectively.

Table 4 shows the testing confusion matrix of SGDM empowered with AlexNet (non-augmented) for predicting kidney cancer at each level. Table 4 depicts the following results: for grade 0, the proposed model correctly predicted 6 positive cancer patients and 12 negative cancer patients and incorrectly predicted 0 positive cancer patients and 0 negative cancer patients; for grade 1, the proposed model correctly predicted 6 positive cancer patients and 1 negative cancer patient and incorrectly predicted 1 positive cancer patient and 0 negative cancer patients; for grade 2, the proposed model correctly predicted 5 positive cancer patients and 12 negative cancer patients and incorrectly predicted 0 positive cancer patients and 1 negative cancer patient.

Table 5 shows the testing confusion matrix of RMSPROP empowered with AlexNet (non-augmented) for predicting kidney cancer at each level. Table 5 depicts the following results: for grade 0, the proposed model correctly predicted 6 positive cancer patients and 12 negative cancer patients correctly and incorrectly predicted 0 positive cancer patients and 0 negative cancer patients; for grade 1, the proposed model correctly predicted 6 positive cancer patients and 10 negative cancer patients and incorrectly predicted 0 positive cancer patients and 2 negative cancer patients; for grade 2, the proposed model correctly predicted 4 positive cancer patients and 12 negative cancer patients and incorrectly predicted 2 positive cancer patients and 0 negative cancer patients.

Table 6 shows the testing confusion matrix of ADAM empowered with AlexNet (non-augmented) for predicting kidney cancer at each level. Table 6 depicts the following results: for grade 0, the proposed model correctly predicted 6 positive cancer patients and 1 negative cancer patient and incorrectly predicted 0 positive cancer patients and 1 negative cancer patient; for grade 1, the proposed model correctly predicted 5 positive cancer patients and 1 negative cancer patient and incorrectly predicted 1 positive cancer patient and 1 negative cancer patient; for grade 2, the proposed model correctly predicted 5 positive cancer patients and 12 negative cancer patients and incorrectly predicted 1 positive cancer patient and 0 negative cancer patients.

Table 7 shows the testing confusion matrix of SGDM empowered with AlexNet (augmented) for predicting kidney cancer at each level. Table 7 depicts the following results: for grade 0, the proposed model correctly predicted 330 positive cancer patients and 659 negative cancer patients but incorrectly predicted 1 positive cancer patient and 0 negative cancer patients; for grade 1, the proposed model correctly predicted 322 positive cancer patients and 660 negative cancer patients and incorrectly predicted 0 positive cancer patients and 8 negative cancer patients; for grade 2, the proposed model correctly predicted 322 positive cancer patients and 651 negative cancer patients and incorrectly predicted 15 positive cancer patients and 2 negative cancer patients.

Table 8 shows the testing confusion matrix of ADAM empowered with AlexNet (augmented) for predicting kidney cancer at each level. Table 8 depicts the following results: for grade 0, the proposed model correctly predicted 330 positive cancer patients and 651 negative cancer patients and incorrectly predicted 1 positive cancer patient and 8 negative cancer patients; for grade 1, the proposed model correctly predicted 321 positive cancer patients and 661 negative cancer patients and incorrectly predicted 1 positive cancer patient and 7 negative cancer patients; for grade 2, the proposed model correctly predicted 322 positive cancer patients and 651 negative cancer patients and incorrectly predicted 15 positive cancer patients and 2 negative cancer patients.

Table 9 shows the testing confusion matrix of RMSPROP empowered with AlexNet (augmented) for predicting kidney cancer at each level. Table 9 depicts the following results: for grade 0, the proposed model correctly predicted 327 positive cancer patients and 649 negative cancer patients and incorrectly predicted 4 positive cancer patients and 10 negative cancer patients; for grade 1, the proposed model correctly predicted 319 positive cancer patients and 663 negative cancer patients and incorrectly predicted 3 positive cancer patients and 5 negative cancer patients; for grade 2, the proposed model correctly predicted 324 positive cancer patients and 648 negative cancer patients and incorrectly predicted 13 positive cancer patients and 5 negative cancer patients.

Table 10 shows the statistical matrix results of the proposed methodology for kidney cancer prediction empowered with IoMT and transfer learning (augmented). The study shows that SGDM had outstanding results throughout training and testing, achieving the highest prediction accuracy and miss-classification rate of 99.20% and 0.80%, respectively. On the other hand, ADAM achieved the second highest prediction accuracy and miss-classification rate of 98.30% and 1.70%, respectively. Finally, RMSPROP achieved a prediction accuracy and miss-classification rate of 98.18% and 1.82%, respectively.

Table 11 shows a comparative analysis of the proposed framework with previous studies. The descriptive analysis shows that Ibrahim et al. [14] achieved a 95% classification accuracy empowered with an LSTM on the miRNA feature dataset. Sheehan et al. [15] achieved an 81% classification accuracy empowered with a DNN on the CT scan image dataset. Ren et al. [16] achieved an 89.7% classification accuracy empowered with an HNN on the clinical feature dataset. Kallenberger et al. [17] achieved an 87% classification accuracy empowered with an RNN on the clinical feature dataset. Vinod et al. [20] achieved a 92.61% classification accuracy empowered with a CNN on the RCC image dataset. Moreau et al. [21] achieved an 89% classification accuracy empowered with a CNN on the Kits19 feature dataset. Lee et al. [26] achieved an 85% classification accuracy empowered with a DNN on the RCC image dataset. Shalski [27] achieved a 92.1% classification accuracy empowered with a vascular tree on the CT scan image dataset. 

We present a comparison of the proposed model with state-of-the-art models in Table 7. It is evident in the table that our proposed methodology has outperformed all current models for osteosarcoma detection in terms of accuracy. The studies cited in [15,26,35] reported low accuracy, and inefficient models were used for classification. Although a few other studies reported promising results [14,16,17,20,21,27], they are unable to ensure the security of patient data, as well as the trained model. Our augmented model achieved a very high accuracy of up to 99.30%. Moreover, our model uses blockchain for the security of data as well as the trained model, while edge computing and fog computing facilitate faster and reliable processing of IoMT-generated data.

This proposed model is beneficial for the assistants of consultants (i.e., a person who organizes the doctor’s appointment—a nontechnical person) and trainees in underdeveloped countries, such as Pakistan, Afghanistan, and Sri Lanka, etc., because, in developing countries, there is a large number of kidney cancer patients of different stages. Therefore, the assistants of consultants (a person who organizes the doctor’s appointment—a nontechnical person) assign appointments on the basis of first come, first serve. Due to this issue, some critical patients were neglected, and some did not survive. In the current scenario in developing countries, it is very difficult to organize appointments on a priority basis. Our system will assist the appointment maker in prioritizing patients based on their test reports, thus saving lives. Our system will also assist and educate the trainees/medical students.

## 6. Conclusions and Future Work

The early diagnosis of kidney cancer empowered with transfer learning and blockchain security is very responsive and can help the health sector apply some major precautions before major health consequences. This study proposed a new IoMT-based kidney cancer prediction framework empowered with transfer learning, which involves some deep learning algorithms and blockchain security technologies. The proposed framework applied SGDM, ADAM, and RMSOROP deep algorithms empowered with transfer learning to obtain some mature results. For the enhancement of results, the proposed framework applied some augmentation and pixel correction techniques. After enhancement and comprehensive model training, the proposed framework achieved 99.2% and 0.8% for test classification accuracy and miss-classification rate, respectively. In this study, all experiments have been descriptively explained. This study plays a major role in the health 5.0 sector for early kidney cancer prediction. Furthermore, in the future, the proposed framework will be expanded using federated learning techniques and machine learning fuzzed model techniques to obtain more mature prediction results.

## Figures and Tables

**Table 1:** Limitations of previous studies.

Study	Model	Data	IoMT	Blockchain Security	Accuracy	Limitations
Ibrahim et al. [14]	LSTM	miRNA (Feature)	NO	NO	95%	More validation for clinical studies, handcrafted features
Sheehan et al. [15]	DNN	CT Scan (Image)	NO	NO	81%	Features issues, imbalance of data
Ren et al. [16]	HNN	Clinical (Feature)	NO	NO	89.7%	Handcrafted feature
Kallenberger et al. [17]	RNN	Clinical (Feature)	NO	NO	87%	Handcrafted feature
Vinod et al. [20]	CNN	RCC (Image)	NO	NO	92.61%	Imbalance data issues
Moreau et al. [21]	CNN	Kits19 (Feature)	NO	NO	89%	Handcrafted features, different stages for more local features
Lee et al. [26]	DNN	RCC (Image)	NO	NO	85%	Performance matrixes should be improved, imbalance data
Shalski [27]	Vascular Tree	CT Scan (Image)	NO	NO	92.1%	Feature selection and data segmentation, imbalance of data

**Table 2:** Pseudocode IoMT-based proposed framework for the prediction of kidney cancer.

Steps	Code
1	Data Source (h_1_, h_2_, h_3_, ………, h_n_)
2	IoMT (Data Source)
3	Data Preprocessing (Augmentation, Pixels Correction, Data Division)
4	Store Preprocessed Data 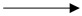 Private Cloud (Blockchain Secured)
5	Transfer Learning 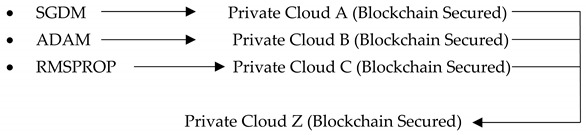
6	Import Test Data 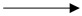 Private Cloud Import Trained Model 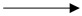 Private Cloud Z
7	Apply Texting (Predict Kidney Cancer)
8	Apply Statistical Matrix

**Table 3:** Training results of AlexNet simulation models empowered with IoMT and blockchain.

AlexNet					
Model	Iterations	Learning Rate	Epoch	CA (%)	MCR (%)
**SGDM**	500	0.001	20	99.8	0.2
**ADAM**				99.00	1.00
**RMSPROP**				98.98	1.02

**Table 4:** Testing confusion matrix of SGDM empowered with AlexNet (non-augmented test data).

Total Samples (18)	Grade 0	Grade 1	Grade 2
**Grade 0**	6	0	0
**Grade 1**	0	6	0
**Grade 2**	0	1	5

**Table 5:** Testing confusion matrix of RMSPROP empowered with AlexNet (non-augmented test data).

Total Samples (18)	Grade 0	Grade 1	Grade 2
**Grade 0**	6	0	0
**Grade 1**	0	6	0
**Grade 2**	0	2	4

**Table 6:** Testing confusion matrix of ADAM empowered with AlexNet (non-augmented test data).

Total Samples (18)	Grade 0	Grade 1	Grade 2
**Grade 0**	6	0	0
**Grade 1**	1	5	0
**Grade 2**	0	1	5

**Table 7:** Testing confusion matrix of SGDM empowered with AlexNet (augmented test data).

Total Samples (990)	Grade 0	Grade 1	Grade 2
**Grade 0**	330	1	0
**Grade 1**	0	322	0
**Grade 2**	0	7	330

**Table 8:** Testing confusion matrix of ADAM empowered with AlexNet (augmented test data).

Total Samples (990)	Grade 0	Grade 1	Grade 2
**Grade 0**	330	0	1
**Grade 1**	0	321	1
**Grade 2**	8	7	322

**Table 9:** Testing confusion matrix of RMSPROP empowered with AlexNet (augmented test data).

Total Samples (990)	Grade 0	Grade 1	Grade 2
**Grade 0**	327	2	2
**Grade 1**	0	319	3
**Grade 2**	10	3	324

**Table 10:** Statistical parameter results for the proposed model for kidney cancer prediction empowered with IoMT and transfer learning (augmented test data).

**SGDM (%)**									
**CA**	**CMR**	**Sen**	**Spec**	**F1**	**PPV**	**NPV**	**FPR**	**FNR**	**LPR**
99.20	0.80	100.00	99.85	99.85	99.70	100.00	0.15	0.00	660.00
**LNR**	**FMI**								
0.00	99.85								
**ADAM (%)**									
**CA**	**CMR**	**Sen**	**Spec**	**F1**	**PPV**	**NPV**	**FPR**	**FNR**	**LPR**
98.30	1.70	99.69	98.95	98.77	97.87	99.85	1.05	0.31	95.13
**LNR**	**FMI**								
0.00	98.77								
**RMSPROP (%)**									
**CA**	**CMR**	**Sen**	**Spec**	**F1**	**PPV**	**NPV**	**FPR**	**FNR**	**LPR**
98.18	1.82	96.14	99.23	97.30	98.48	98.03	0.77	3.86	125.56
**LNR**	**FMI**								
0.04	97.30								

**Table 11:** Comparative analysis of the proposed framework with previous studies.

Study	Model	Dataset	IoMT	Blockchain Security	Accuracy
Ibrahim et al. [14]	LSTM	miRNA (Feature)	NO	NO	95%
Sheehan et al. [15]	DNN	CT Scan (Image)	NO	NO	81%
Ren et al. [16]	HNN	Clinical (Feature)	NO	NO	89.7%
Kallenberger et al. [17]	RNN	Clinical (Feature)	NO	NO	87%
Vinod et al. [20]	CNN	RCC (Image)	NO	NO	92.61%
Moreau et al. [21]	CNN	Kits19 (Feature)	NO	NO	89%
Lee et al. [26]	DNN	RCC (Image)	NO	NO	85%
Shalski [27]	Vascular Tree	CT Scan (Image)	NO	NO	92.1%
Benchmark [35]	ResNet Custom	Biopsy (Image)	No	No	79%
Benchmark [35]	VGG Net	Biopsy (Image)	No	No	20%
**The Proposed Model (Augmented Test Data)**	**Transfer Learning (SGDM, ADAM, RMSPROP)**	**Biopsy (Image)**	**Yes**	**Yes**	**99.2%**
**The Proposed Model (NonAugmented Test Data)**	**Transfer Learning (SGDM, ADAM, RMSPROP)**	**Biopsy (Image)**	**Yes**	**Yes**	**93.75%**

## Data Availability

The simulation files/data used to support the findings of this study are available from the corresponding author upon request.
